# Systematic identification and comparative analysis of lysine succinylation between the green and white parts of chimeric leaves of *Ananas comosus* var. *bracteatus*

**DOI:** 10.1186/s12864-020-6750-6

**Published:** 2020-06-03

**Authors:** Meiqin Mao, Yanbin Xue, Yehua He, Xuzixing Zhou, Fatima Rafique, Hao Hu, Jiawen Liu, Lijun Feng, Wei Yang, Xi Li, Lingxia Sun, Zhuo Huang, Jun Ma

**Affiliations:** 1 College of Landscape Architecture, Sichuan Agricultural University, Chengdu, China; 2 Horticultural Biotechnology College, South China Agricultural University, Guangzhou, China

**Keywords:** *Ananas comosus* var., *bracteatus*, Lysine succinylation, Chimeric leaves, CAM photosynthesis, Energy metabolism

## Abstract

**Background:**

Lysine succinylation, an important protein posttranslational modification (PTM), is widespread and conservative. The regulatory functions of succinylation in leaf color has been reported. The chimeric leaves of *Ananas comosus* var. *bracteatus* are composed of normal green parts and albino white parts. However, the extent and function of lysine succinylation in chimeric leaves of *Ananas comosus* var. *bracteatus* has yet to be investigated.

**Results:**

Compared to the green (Gr) parts, the global succinylation level was increased in the white (Wh) parts of chimeric leaves according to the Western blot and immunohistochemistry analysis. Furthermore, we quantitated the change in the succinylation profiles between the Wh and Gr parts of chimeric leaves using label-free LFQ intensity. In total, 855 succinylated sites in 335 proteins were identified, and 593 succinylated sites in 237 proteins were quantified. Compared to the Gr parts, 232 (61.1%) sites in 128 proteins were quantified as upregulated targets, and 148 (38.9%) sites in 70 proteins were quantified as downregulated targets in the Wh parts of chimeric leaves using a 1.5-fold threshold (*P* < 0.05). These proteins with altered succinylation level were mainly involved in crassulacean acid metabolism (CAM) photosynthesis, photorespiration, glycolysis, the citric acid cycle (CAC) and pyruvate metabolism.

**Conclusions:**

Our results suggested that the changed succinylation level in proteins might function in the main energy metabolism pathways—photosynthesis and respiration. Succinylation might provide a significant effect in the growth of chimeric leaves and the relationship between the Wh and Gr parts of chimeric leaves. This study not only provided a basis for further characterization on the function of succinylated proteins in chimeric leaves of *Ananas comosus* var. *bracteatus* but also provided a new insight into molecular breeding for leaf color chimera.

## Background

PTM is an important regulator of protein activities and conformations and of protein-protein interactions (PPIs) that modulate many biological processes [1, 2]. Over 450 PTMs have been identified to date, and methylation, acetylation, propionylation, ubiquitination, phosphorylation, malonylation, succinylation and crotonylation are common PTMs [3]. PTMs regulate protein activities and conformations by adding new functional groups to amino acid residue. Lysine succinylation, a new lysine acylation, introduces succinyl group (−CO-CH_2_-CH_2_-CO-) into protein. Succinyl group changes the charge on the modified residues from + 1 to − 1, and the charge changes were higher than charge changes (+ 1 to 0) which is due to acetylation [4]. In turn, this will result in greater changes in structure and function of succinylated protein. Therefore, lysine succinylation may regulate other novel and complex cellular activities [5]. Succinylation was first reported in histone proteins, and it therefore can function in regulating gene expression through effects on chromatin structure [6]. Lysine succinylation has been studied in diverse organisms and tissues [7, 8], and succinylated proteins are abundant in mitochondrial metabolism, including the CAC, amino acid degradation and fatty acid metabolism [9].

*Ananas comosus* var. *bracteatus*, which belongs to the Bromeliaceae family, is an herbaceous perennial monocot. Owing to its red fruits, it is a good tropical ornamental plant [10]. Based on observations made by ordinary microscopy, the chimeric leaves are composed of the normal green cells and albino white cells, and the albino white cells have no intact chloroplasts (Additional file 1: Figure S1). Therefore, the chimeric leaves of *Ananas comosus* var. *bracteatus* are excellent materials for studying pigment biosynthesis, photosynthesis mechanism, nuclear-plastid genome and other related metabolic processes. A great many of genes have been studied to analyze the mechanism of chimeric leaves formation and growth in *Ananas comosus* var. *bracteatus* [10–13]. However, the PTM-mediated regulatory mechanism in chimeric leaves of *Ananas comosus* var. *bracteatus* is largely unknown. Western blot experiments were performed, which confirmed the existence of acetylation and succinylation in chimeric leaves of *Ananas comosus* var. *bracteatus* (Additional file 2: Figure S2). The level of acetylation and succinylation in the Wh parts of chimeric leaves was increased. And lysine succinylation has been identified as a likely candidate for the regulation of leaf color through modulating multiple metabolic pathways and coordination of different metabolic pathways [14–16]. Therefore, revealing the lysine succinylation profile in *Ananas comosus* var. *bracteatus* may be important for the study of regulatory mechanisms in the formation and growth of chimeric leaves. We performed the first proteomic study on lysine succinylation in *Ananas comosus* var. *bracteatus.* Succinylated sites and proteins in *Ananas comosus* var. *bracteatus* were systematically identified, and the differences in the succinylation profiles between the Wh and Gr parts of chimeric leaves were also reported. Overall, a total of 855 succinylated sites in 335 proteins with diverse cellular localizations and biological processes were identified, and 380 differentially expressed lysine succinylation sites were quantified. The succinylation level was increased in the Wh parts of chimeric leaves. Finally, the correlation between succinylation level and multiple metabolic processes including CAM photosynthesis, photorespiration, glycolysis, the CAC and pyruvate metabolism were discussed. In this study, therefore, we provided a new insight into succinylation on formation and growth of chimeric leaves*.*

## Results and discussion

### Changes in the content of starch, malate and soluble sugar in the Wh parts of chimeric leaves

**Fig. 1:**
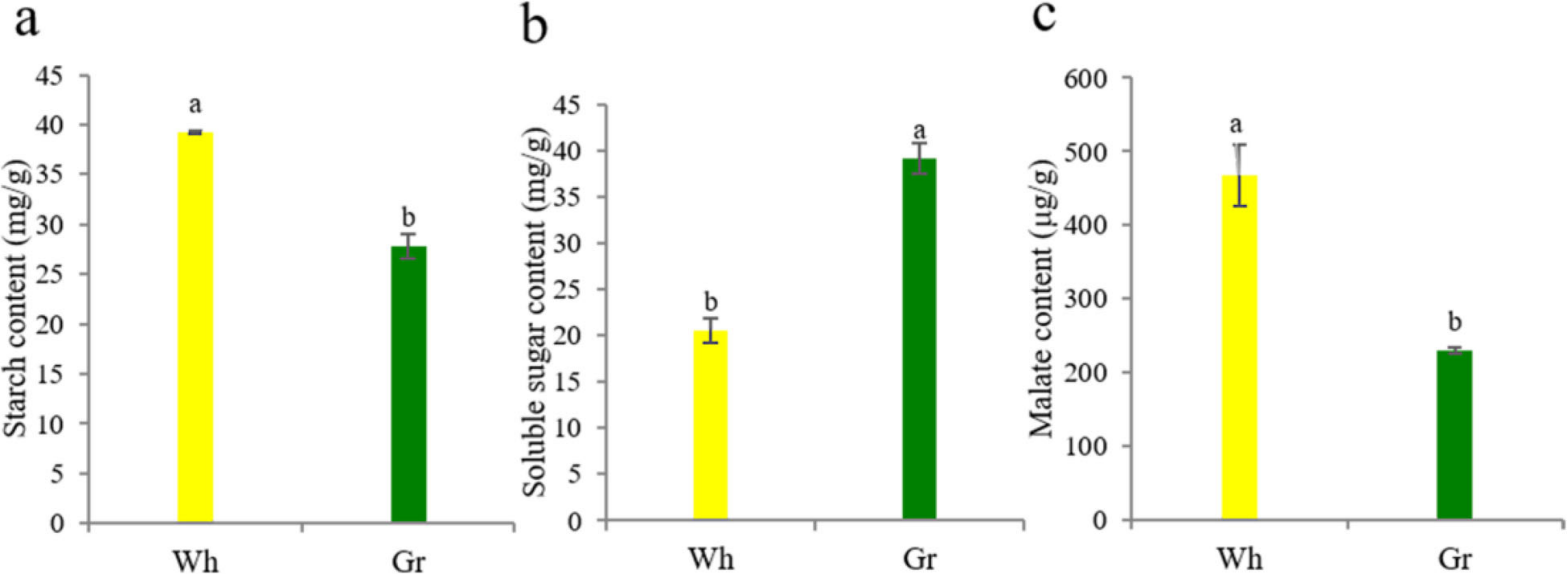
Measurement of starch, soluble sugar and malate content between the two parts of chimeric leaves in *Ananas comosus* var. *bracteatus*. **a** Starch content. **b** Soluble sugar content. **c** Malate content. Standard error of the mean for three repetitions is represented by the error bars. The different letters above the bars indicate the significant difference at *P* < 0.05 between two parts. Wh: white parts; Gr: green parts

Plant leaf albino is an obvious and common chlorophyll deficient mutation, which affects plant growth by changing physiological and biochemical levels [17]. The chimeric leaves in *Ananas comosus* var. *bracteatus* are composed of the normal green parts and albino white parts. Compared with the Gr parts, the Wh parts had higher starch content and lower soluble sugar content (*P* < 0.05; Fig. 1a and b). Some study showed that lower photosynthetic rate is due to accumulated starch content and decreased soluble sugar content [18]. In addition, the Wh parts had higher malate content (*P* < 0.05; Fig. 1c). Malate is the initial product of CO_2_ fixation in CAM plant, and also is the respiratory substrate for ATP production in mitochondria [19]. Our results suggested that photosynthetic activity and respiratory property were altered between the two parts.

### The proteome profile was altered in the Wh parts of chimeric leaves

Compared to the Gr parts, 805 proteins were upregulated and 457 proteins were downregulated in the Wh parts of chimeric leaves using a 1.5-fold threshold (*P* < 0.05; Additional file 3: Table S1). Many of the upregulated proteins were enriched in the spliceosome, ribosome, mRNA surveillane pathway and RNA degradation (Additional file 4: Figure S3). Therefore, the different manner of gene regulation might exist between the Wh and Gr parts of chimeric leaves. Whereas a large portion of downregulated protein were highly enriched in photosynthesis, glycolysis, oxidative phosphorylation and citrate cycle (Additional file 4: Figure S3). These results suggested that the function of photosynthesis and energy metabolism might be suppressed in the Wh parts of chimeric leaves. This is accordance with our comparative proteomic data studied previously [13]. Furthermore, the overlap in differentially expressed proteins and proteins with differentially expressed lysine succinylation sites was studied. There were 51 proteins with consistent changes between succinylation levels and protein abundance, whereas 30 proteins demonstrated opposing changes (Additional file 5: Table S2).

### The level of succinylation in the Wh parts of chimeric leaves was increased

**Fig. 2:**
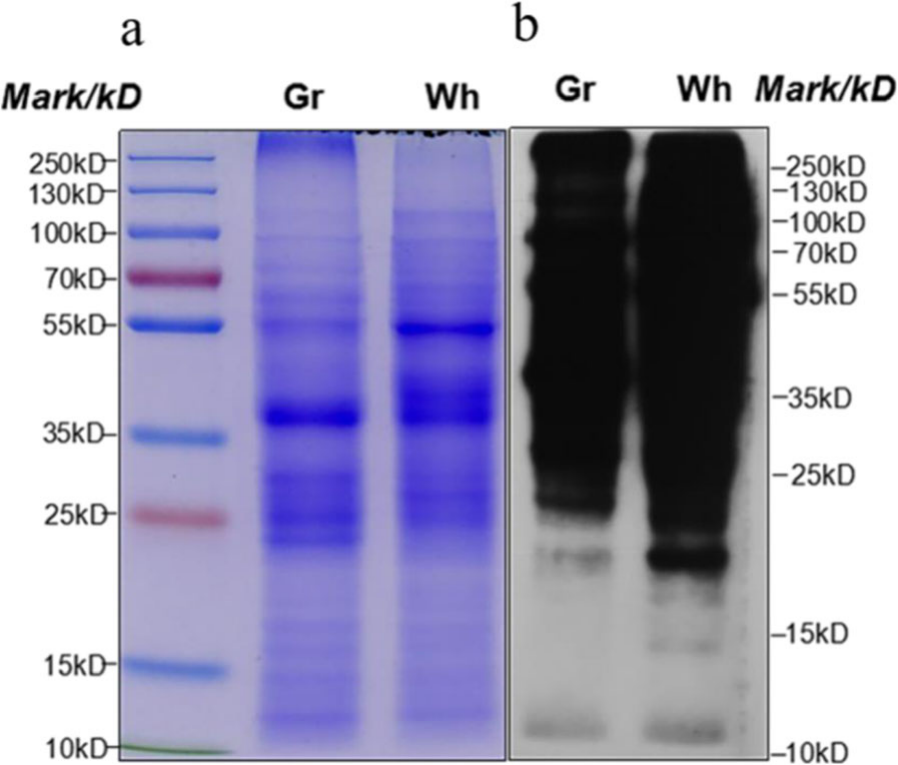
Western blot analysis of the succinylation levels between the two parts of chimeric leaves in *Ananas comosus* var. *bracteatus*. **a** SDS-PAGE stained with coomassie blue. **b** Western blot of protein succinylation. Same amount of proteins (20 μg per lane) were loaded as in each panel. Wh: white parts; Gr: green parts

**Fig. 3:**
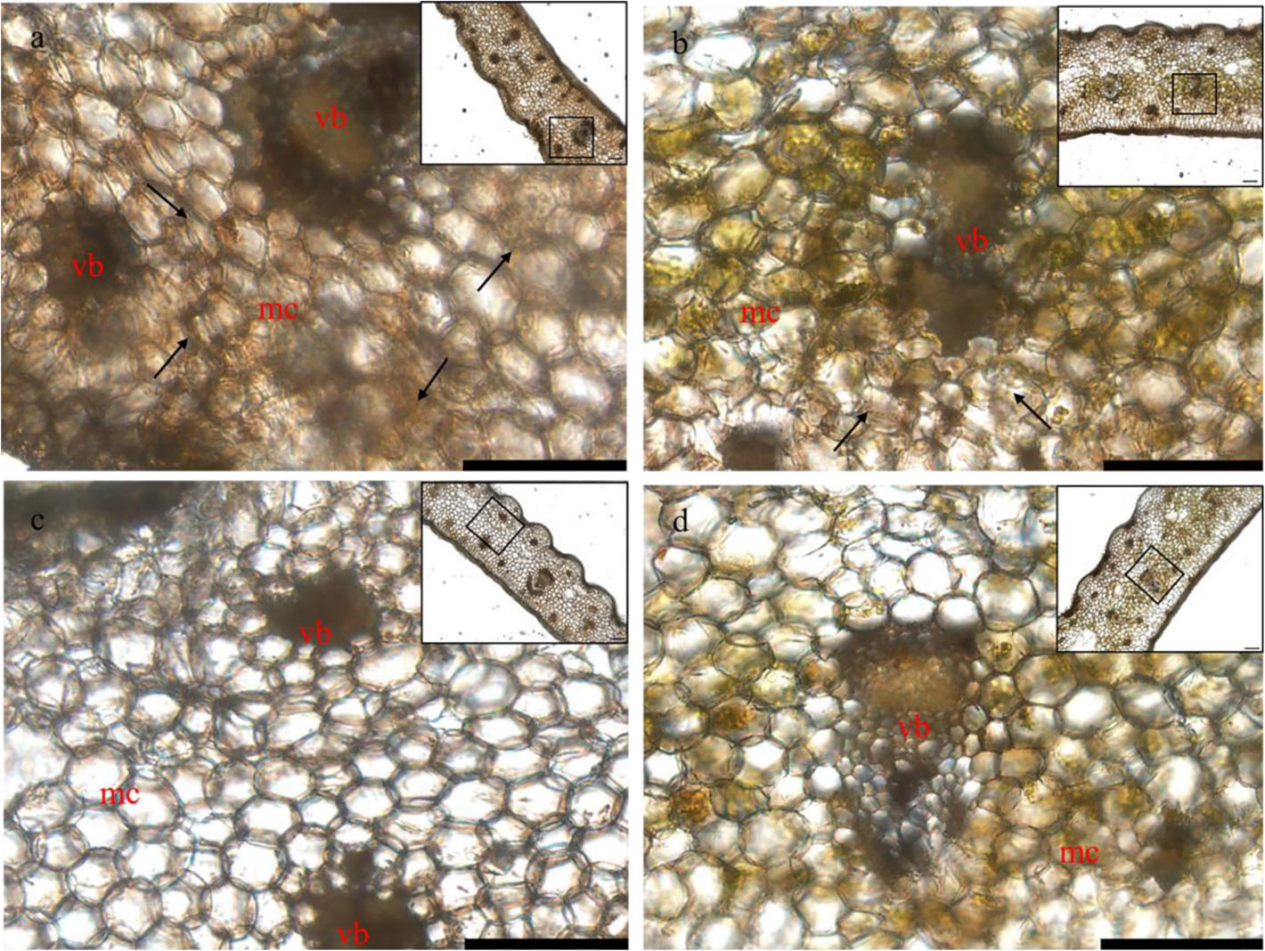
Immunohistochemistry analysis of the succinylation levels between the two parts of fresh chimeric leaves in *Ananas comosus* var. *bracteatus.***a** Immunohistochemistry analysis of the white (Wh) parts of chimeric leaves against antisuccinyllysine antibody. **b** Immunohistochemistry analysis of the green (Gr) parts of chimeric leaves against antisuccinyllysine antibody. **c** and **d** Negative control of the Wh parts (**c**) and Gr parts (**d)** of chimeric leaves against PBS. The black frame indicates an observation range, which is composed of vascular bundle (vb) and mesophyll cell (mc) surrounding the vb. The positive staining signal is brown and the black arrow indicates the positive region. Scale bar = 100 μm (in a-d)

To obtain an overview of the extent of lysine succinylation in chimeric leaves of *Ananas comosus* var. *bracteatus*, we performed Western blot analysis using lysine succinylation-specific pan-antibodies. Lysine succinylation was observed on a great many of proteins with varying molecular masses in both green and white leaf samples (Fig. 2). These results suggested that lysine succinylation was abundant in chimeric leaves of *Ananas comosus* var. *bracteatus*. Notably, succinylation level in the Wh parts of chimeric leaves was significantly higher than that of the Gr parts in Western blot. In order to analyze the succinylation level in situ, immunohistochemistry analysis of the freehand sections of the Wh and Gr parts of chimeric leaves were carried out. Compared to negative control (Fig. 3c, d), both the Wh parts (Fig. 3a) and Gr parts (Fig. 3b) of chimeric leaves possessed brown positive signal. Furthermore, the staining of lysine succinylation in the Wh parts of chimeric leaves was stronger than that of the Gr parts. These results indicated that the succinylome level in the Wh parts of chimeric leaves was increased.

### Proteome-wide analysis of lysine-succinylated peptides and proteins in *Ananas comosus* var. *bracteatus*

**Fig. 4:**
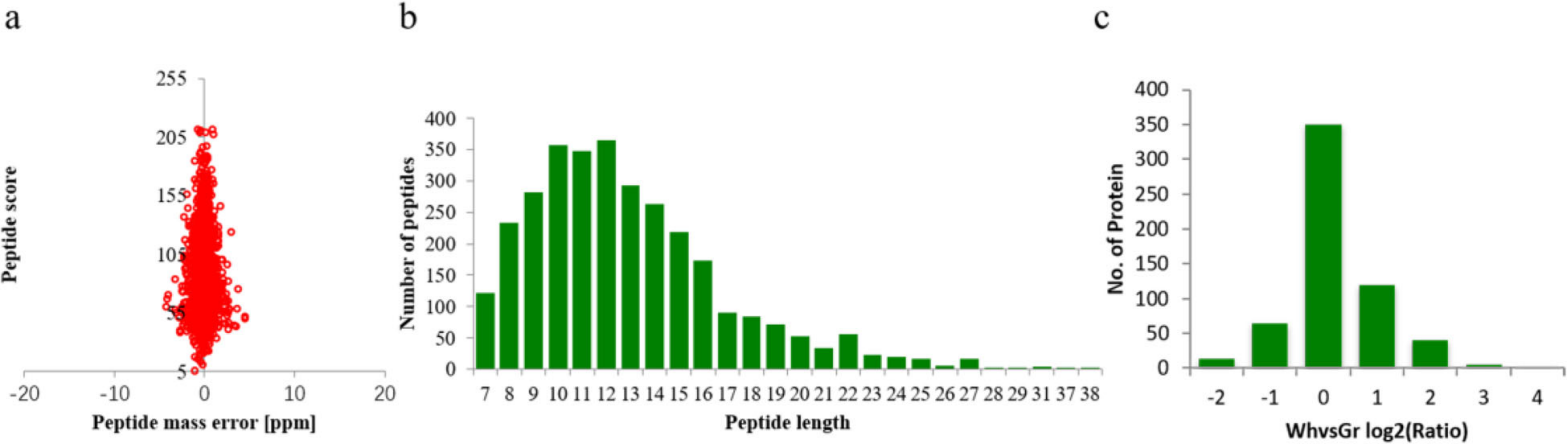
The basic information of LC-MS/MS data. **a** Mass error distribution of all identified peptides. **b** Peptide length distribution. **c** Succinylome quantitative data distribution. Wh: white parts; Gr: green parts

The protein succinylation in the Gr and Wh parts of chimeric leaves was revealed by combining with anti-succinyllysine antibody-dependent enrichment and high-resolution liquid chromatographytandem mass spectrometry (LC-MS/MS). We checked the mass error of all the identified peptides to assess the accuracy of MS data. As shown in Fig. 4a, the mass error of all the identified peptides was near zero, which indicates that the reliability of the MS data fit the requirement. With regard to peptide length, most peptides were distributed between 8 and 16, which suggests that sample preparation met the standards (Fig. 4b). And succinylome quantitative data distribution was shown in Fig. 4c.

**Fig. 5:**
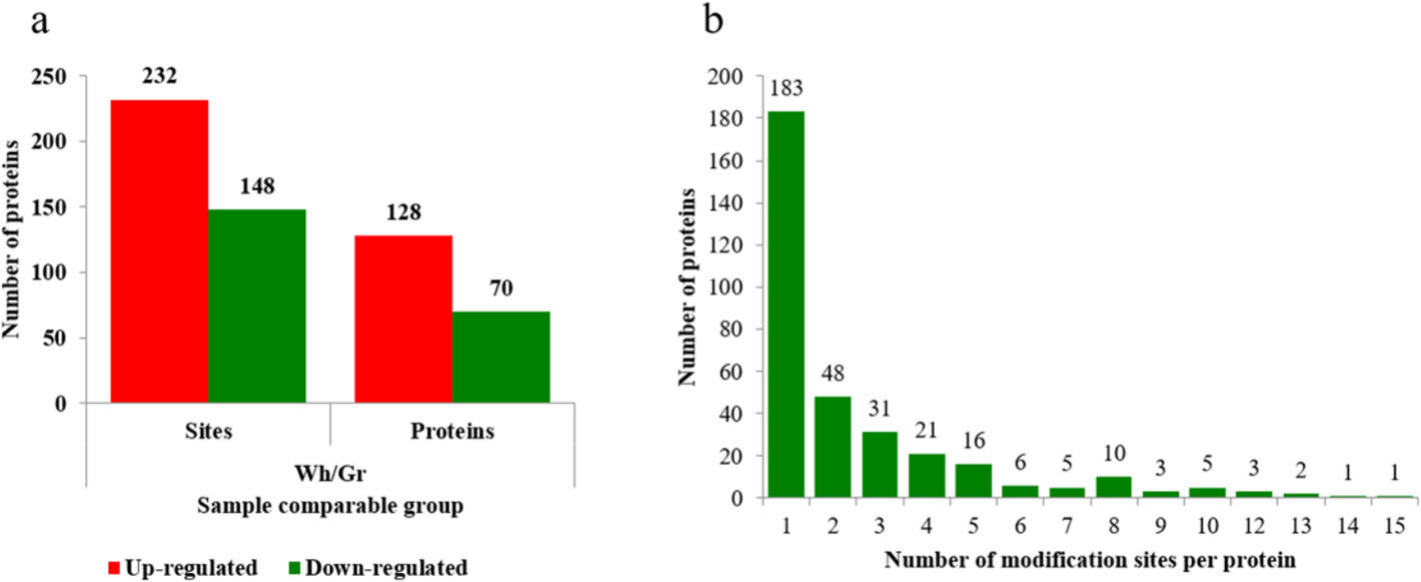
Succinylation profile between the two parts of chimeric leaves in *Ananas comosus* var. *bracteatus*. **a** Number of differentially expressed sites and proteins. **b** Distribution of succinylated proteins based on number of succinylation. Wh: white parts; Gr: green parts

After LC-MS/MS analysis and database search, a total of 855 succinylated sites in 335 proteins were identified, and 593 succinylated sites in 237 proteins were accurately quantified. Compared to the Gr parts, 232 (61.1%) sites in 128 proteins were quantified as upregulated targets, and 148 (38.9%) sites in 70 proteins were quantified as downregulated targets in the Wh parts of chimeric leaves using a 1.5-fold threshold (*P* < 0.05; Fig. 5a; Additional file 6: Table S3). These results showed that global succinylation level was increased in the Wh parts of chimeric leaves. This is accordance with the Western blot and immunohistochemistry analysis results.

Previous studies showed that various succinylated proteins have been identified in bacteria [9], fungi [20], protozoans [21] and mammalian cells [14, 22]. However, only nine succinylome studies have been reported in plants. The number of succinylated proteins in rice [5] and tea [16] is almost eight times and six times more than that in *Ananas comosus* var. *bracteatus*, respectively. But the number of succinylated proteins in *Ananas comosus* var. *bracteatus* was much higher than that in strawberry stigmata [23], common wheat [24], rice seeds [25], tomato [26], *Taxus*×*media* [27], *Brachypodium distachyon* [28], *Dendrobium officinale* [29]. In physiological level, different species and tissues may possess differential profile of succinylation. In technical level, sample preparation, method, number of proteins in the databases varied among researches may result in the different succinylated profile. Notably, 5 succinylation sites were found on histone proteins in *Ananas comosus* var. *bracteatus*, including 2 sites on H2B.1, 2 sites on H3.3 and 1 site on H4. Lysine succinyltion found in histone represents an evolutionarily conserved histone mark in eukaryotic [6]. And modification at different locations or different PTMs at the same histone site can be associated with very different transcriptional programs [6].

The number of succinylated sites in the identified proteins was counted in this study (Fig. 5b). Of the succinylated proteins, 54.6% (183/335) had only one succinylated site, 14.3% (48/335) possessed two succinylated sites, 9.3% contained three succinylated sites, and the remaining were modified on four or more lysine residues. Each succinylated protein had 2.55 (855/335) succinylated sites on average. Notably, ribulose bisphosphate carboxylase (RuBisCO) large chain, which is the protein with the most succinylated sites in chimeric leaves of *Ananas comosus* var. *bracteatu*, possessed 15 succinylated sites. Similarly, the large chain of RuBisCO is also extensively succinylated in rice leaves, containing 16 independent succinyl-lysine residues [5].

### Functional annotation and subcellular localization of the succinylated proteins

**Fig. 6:**
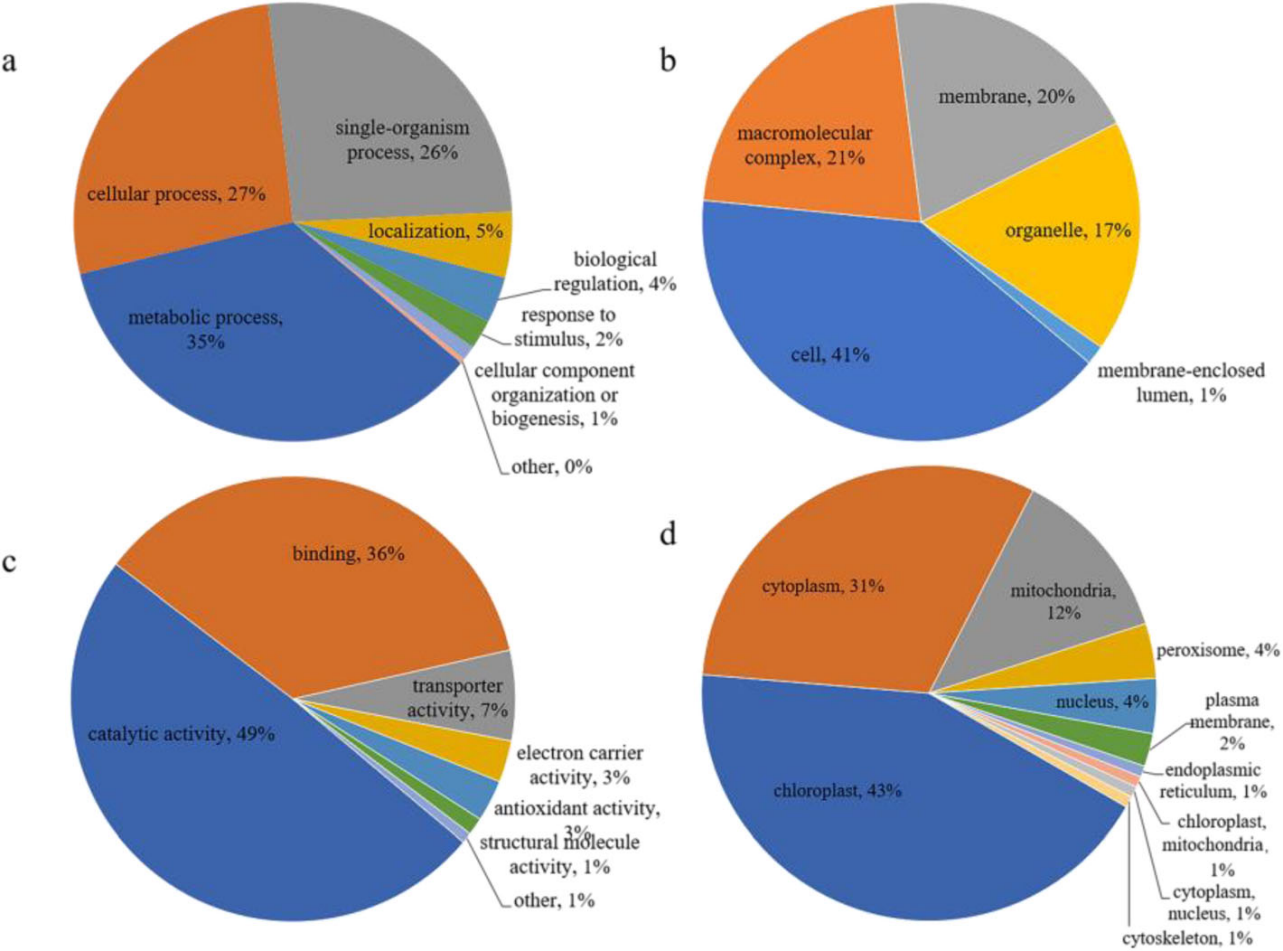
Pie charts showing the functional classification of succinylated proteins. **a** Classification of the succinylated proteins based on biological process. **b** Classification of the succinylated proteins based on cellular component. **c** Classification of the succinylated proteins based on molecular function. **d** Subcellular localization of the succinylated proteins

Using Gene Ontology (GO) functional classification analysis, the potential role of succinylation in chimeric leaves of *Ananas comosus* var. *bracteatus* was studied. In biological process (Fig. 6a), the three largest groups of succinylated proteins were involved in metabolic process (35%), followed by cellular process (27%) and single-organism process (26%). This is accordance with other plants [25, 26, 28], suggesting that this distribution pattern is not novel at all. In cellular component (Fig. 6b), most succinylated proteins were located in the cell (41%), macromolecular complex (21%), membrane (20%) and organelle (17%). In molecular function (Fig. 6c), we found that the largest group of succinylated proteins (49%) was related to catalytic activities, suggesting that the succinylation enzyme may affect biological processes. The second largest group (36%) possesses binding activities, which means succinylation may work in DNA transcription and PPIs. So, in conclusion, lysine succinylation may affect multiple biological processes in chimeric leaves of *Ananas comosus* var. *bracteatus* by changing the molecular functions of proteins in diverse cellular components.

The subcellular localizations of the identified proteins were also predicted. Generally, succinylation is highly concentrated in mitochondria because the succinyl-CoA and succinate formed via the CAC and odd numbered fatty acid oxidation primarily accumulates in the mitochondrial matrix [3]. For example, 70% of succinylated proteins mainly exist in the mitochondria in mouse liver cells [22]. In addition to non-enzymatic succinylation by succinyl CoA, succinylation can be mediated by in an α-ketoglutarate-dependent manner [3]. The oxoglutarate dehydrogenase (OGDH), which is a component of the α-ketoglutarate dehydrogenase (KGDH) complex, can serve as a succinyltransferase [3]. Some study indicated that the α-KGDH complex is much greater effective than succinyl-CoA owing to the catalysis of the OGDH [30]. In this study, most succinylated proteins were located in the chloroplast, cytoplasm, mitochondria and nucleus, accounting for 47, 23, 16 and 7% of all the identified proteins, respectively (Fig. 6d). It revealed that lysine succinylation can exist in outside of mitochondria. One possibility is that a functional α-KGDH complex exist in outside of mitochondria. Some study indicated that the component and activity of α-KGDH complex can be readily measured in cytosolic fractions [29]. And experiments have shown that α-KGDH complex can be localized in the nucleus [31]. But whether it is localized in the chloroplast has not been experimentally proven. A second, but unlikely, possibility is that succinyl-CoA is transported from the mitochondria. A third possibility is that an alternative succinyltransferase depending on α-ketoglutarate manner exists in outside of mitochondria. But other explanations are possible. Notably, the number of succinylated chloroplast proteins was much higher than that of succinylated mitochondrial proteins in this study. This is accordance with other plants [24, 27]. The detection of succinylation sites is biased to occur on more abundant proteins [22]. Therefore, a larger number of succinylation sites can be identified on chloroplast proteins that accounted for a large proportion of total protein in plants.

### Analysis of succinylated lysine sequence motifs

**Fig. 7:**
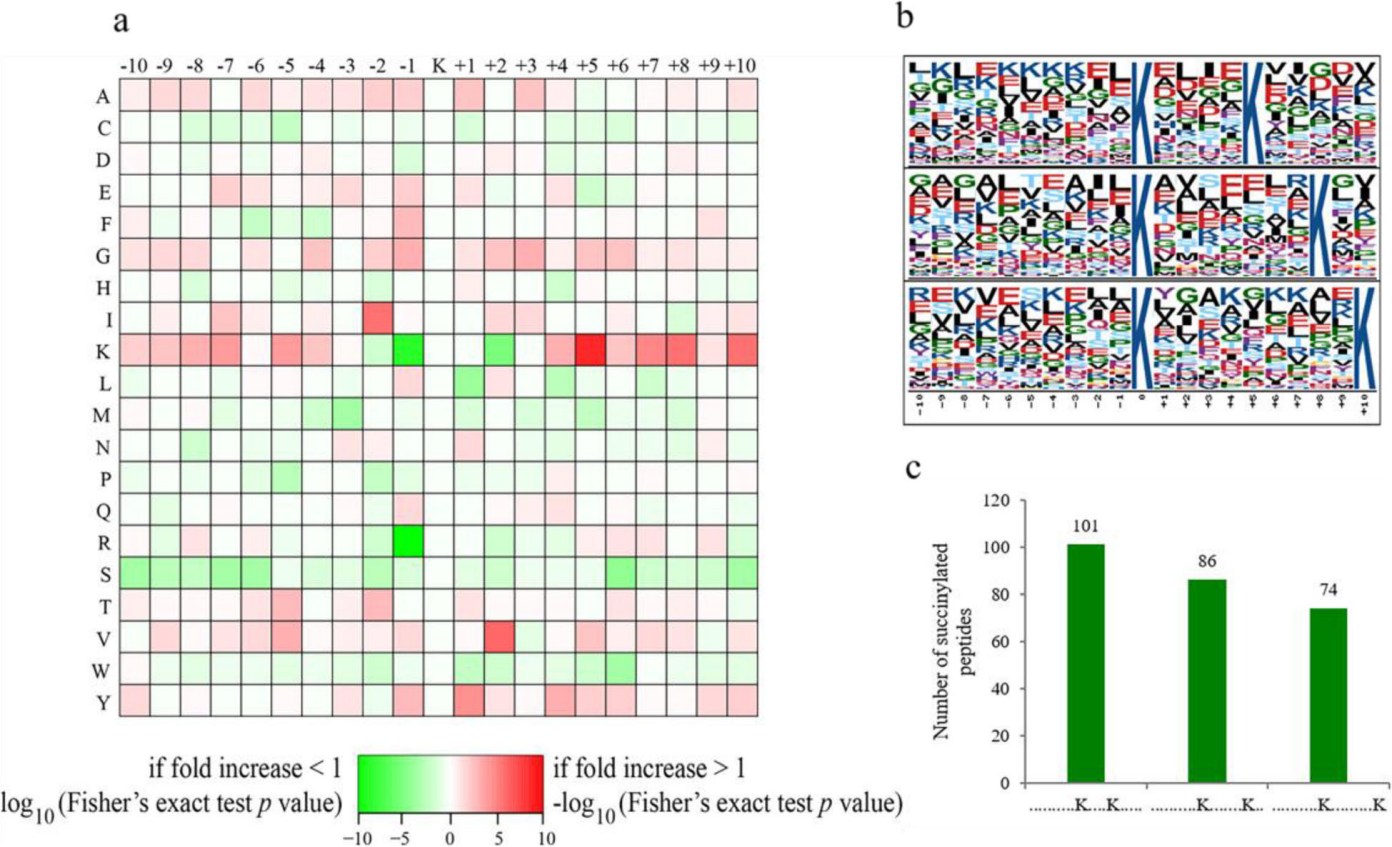
Properties of lysine succinylated peptides. **a** Heat map of the amino acid compositions of the succinylated sites. **b** Succinylation sequence motifs for ±10 amino acids around the lysine succinylation sites. **c** Number of peptides containing each of the conserved motifs

The frequency of different amino acids around the succinylated lysine from − 10 to + 10 was measured, which can investigate the nature of succinylated sites in chimeric leaves of *Ananas comosus* var. *bracteatus*. The frequency of lysine (K) at + 5 was highest (Fig. 7a). Using the motif-x program, the sequence motifs in all the identified peptides were identified. Three conserved motifs were identified from 855 succinylated sites, namely, K^su^(X9) K, K^su^(X7) K and K^su^(X4) K (Ksu indicates the succinylated lysine, and X indicates a random amino acid residue) (Fig. 7b), and these motifs exhibited different abundances (Fig. 7c). Among these motifs, K^su^(X4) K and K^su^(X7) K were previously identified in other plant species [16, 18, 19, 21–28, 32]. Notably, K^su^(X7) K was also observed in the marine bacterium [33], indicating that some motifs might be conservative between plant and bacteria.

### The succinylome profile was changed in the Wh parts of chimeric leaves

**Fig. 8:**
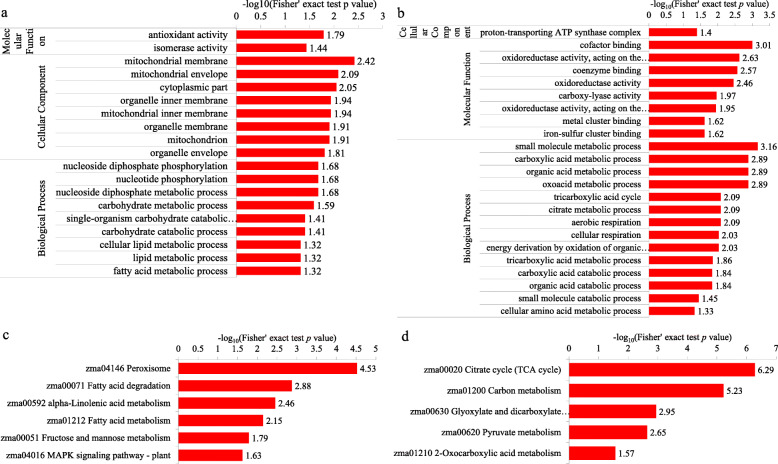
Functional enrichment analysis of proteins with upregulated and downregulated Ksu sites in the white (Wh) parts. **a** and **b** GO-based enrichment analysis of proteins with up-regulated and down-regulated Ksu sites. **c** and **d** KEGG pathway-based enrichment analysis of proteins with upregulated and downregulated Ksu sites

To explore the role of succinylation in the formation and growth of chimeric leaves in *Ananas comosus* var. *bracteatus*, we analyzed the proteins which possess differentially expressed lysine succinylation sites between the Wh and Gr parts of chimeric leaves using GO annotation and Kyoto encyclopedia of genes and genomes (KEGG) pathway enrichment analysis (*P*<0.05; Fig. 8). Briefly, in molecular function enrichment analysis, proteins with upregulated Ksu sites in the Wh parts were associated with antioxidant activity and isomerase activity. For example, the intensity of all Ksu sites in catalase-1 and superoxide dismutase [Cu-Zn] was markedly increased in the Wh parts. It is possible that a higher level of succinylation maintains cellular redox homeostasis in the Wh parts of chimeric leaves through altering the activities of antioxidant enzymes. Conversely, proteins with downregulated Ksu sites in the Wh parts were associated with oxidoreductase activity and binding activity. In detail, these proteins with downregulated Ksu sites mainly are core enzyme and coenzyme in the CAC and mitochondrial electron transport chain (ETC). In cellular component enrichment analysis, we found that proteins with upregulated Ksu sites in the Wh parts were highly located at mitochondria. Mitochondria is power house of eukaryotic cells, which can fuel metabolism with ATP to maintain the movement and growth of organism [34]. Conversely, proteins with downregulated Ksu sites in the Wh parts were enriched in the ATP synthase complex. ATP synthase is a key enzyme in photophosphorylation and oxidative phosphorylation, affecting the production of ATP required for cell life activities. These results suggested photosynthetic activity and respiratory properties were altered in the Wh parts of chimeric leaves. It might result from the downregulated succinylation of proteins associated with ATP synthase complex. In the biological process enrichment analysis, differentially changed succinylated proteins were enriched in 23 processes, particularly processes involved in metabolism and energy generation. KEGG pathway enrichment analysis of proteins whose succinylation level changed was carried out. The protein-processing pathways in the peroxisome, fatty acid degradation, alpha-linolenic metabolism, fatty acid metabolism, fructose and mannose metabolism, and plant MAPK signaling pathway were enriched among the proteins with upregulated Ksu sites in the Wh parts. Whereas upregulated proteins in the Wh parts were mainly enriched in spliceosome and ribosome through previous study [13]. Proteins with downregulated Ksu sites in the Wh parts were enriched in pathways involving the CAC, carbon metabolism, glyoxylate metabolism, dicarboxylate metabolism, pyruvate metabolism, and 1-oxocarboxylic acid metabolism. And previous study has shown that lots of downregulated proteins in the Wh parts were enriched in photosynthesis and respiration [13], which indicated that protein abundant and succinylation level may work together to regulate photosynthesis and respiration in chimeric leaves. These results suggested that the changed succinylation level may be a candidate regulator to metabolism- and energy-related processes of chimeric leaves in *Ananas comosus var. bracteatus*.

### Succinylated proteins involved in CAM photosynthesis in the Gr and Wh parts of chimeric leaves

Photosynthesis, which provides chemical energy for maintaining plant life, plays important roles in plant metabolic processes [35]. In this study, the Wh parts of chimeric leaves had higher starch content and lower soluble sugar content, which suggested the Wh parts take a low photosynthetic rate and may act as a photosynthetic product sink. Previous studies have shown that lysine succinylation is prevalent in various photosynthetic organisms [8]. In this study, six succinylated proteins involved in photosynthesis were identified only in the Gr parts of chimeric leaves, including three subunits in photosystem I (PsaC, PsaD, PsaF), two subunits in photosystem II (PsbB, PsbP) and light-harvesting complex II chlorophyll a/b binding protein 2 (Lhcb2). Furthermore, succinylation level on cytochrome b6-f complex iron-sulfur subunit (PetC) was downregulated and that on photosystem II oxygen-evolving enhancer protein 1 (PsbO) was upregulated in the Wh parts of chimeric leaves. In detail, the succinylation level of the K162 site in PsbO was upregulated about eight times. PsbO is the largest extrinsic subunit of PSII, which plays critical roles in oxygen evolution reaction [36]. In cyanobacterium, PsbO succinylation can hinder correct interactions between PsbO and other PSII subunits by the conformation changes in the head domain of PsbO, leading to a negative regulation of oxygen evolution [8]. However, the Ksu sites in PsbO of *Ananas comosus* var. *bracteatus* differed from cyanobacterium, it may work differently in regulating photosynthesis. The increase (decrease) of succinylation level of the light harvesting proteins, PSI and PSII proteins may work in the decrease of the photosynthetic rate in the Wh parts of the chimeric leaves.

**Fig. 9:**
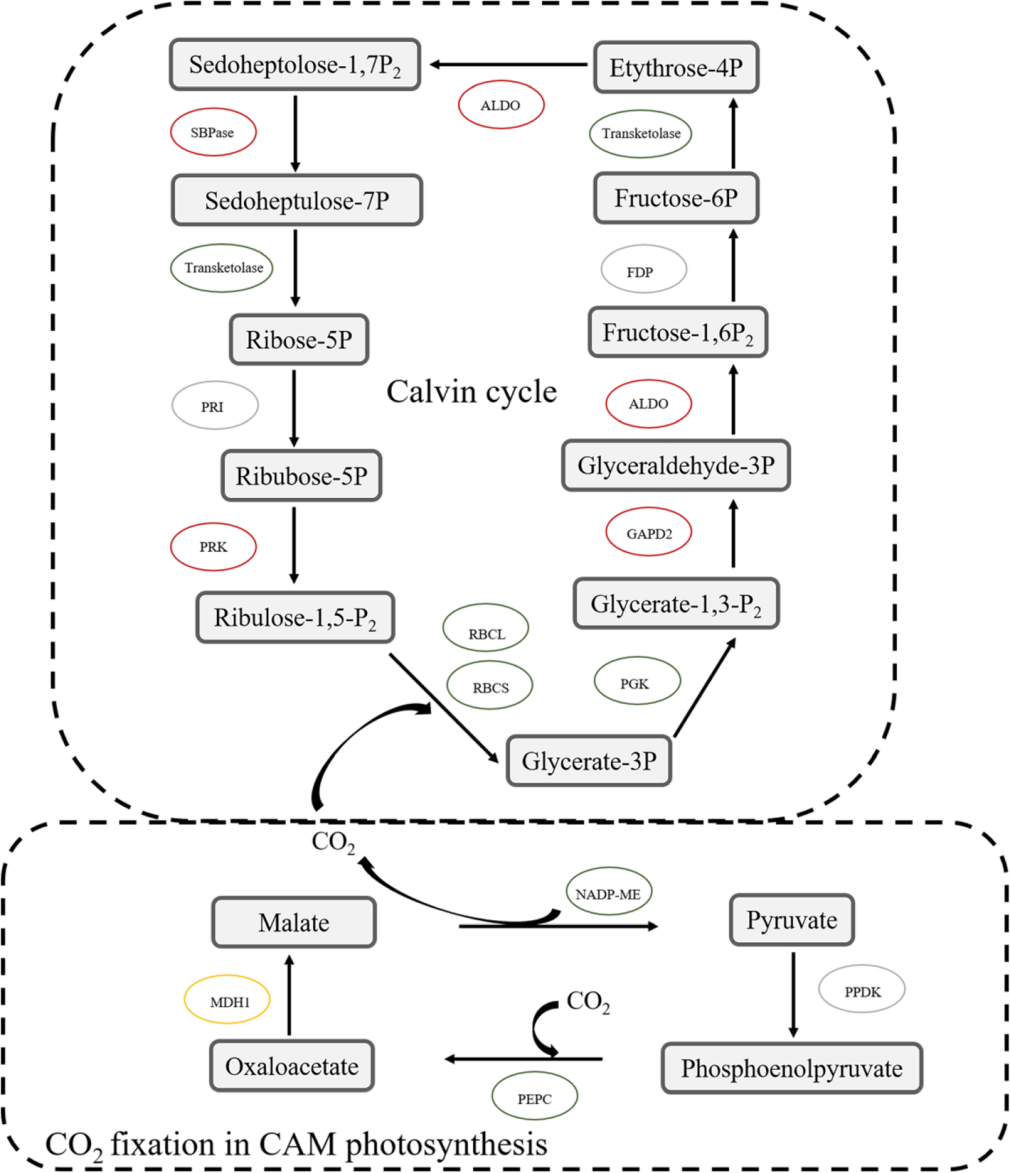
Succinylated enzymes involved in the Calvin cycle. Red oval represents proteins possessed upregulated sites in the white (Wh) parts, green oval represents proteins possessed down regulated sites in the white (Wh) parts, yellow oval represents proteins both possessed upregulated and downregulated sites in the white (Wh) parts, grey oval represents proteins with no Ksu site

Pineapple is a typical CAM plant. The hallmark of CAM photosynthesis is the conversion of CO_2_ into malate at night. In this study, some enzymes involved catalyzing the conversion of CO_2_ into malate were identified as succinylated proteins, including phosphoenolpyruvate carboxylase (PEPC), NAD-dependent malic enzyme (NADP-ME) and malate dehydrogenase (MDH1) (Fig. 9). In detail, succinylation level in PEPC and NADP-ME were upregulated in the Wh parts of chimeric leaves. But MDH1 not only possessed an upregulated site but also a downregulated site in the Wh parts. Enzymatic assays showed that the activity of NADP-ME in the Wh parts of chimeric leaves was significantly higher than that in the Gr parts (*P* < 0.05; Additional file 7: Figure S4), but the Wh and Gr parts of chimeric leaves had no significant difference in the activity of PEPC and MDH1 (Additional file 7: Figure S4). NADP-ME in the CAM plants plays a key role in photosynthesis by providing CO_2_ for fixation in the Calvin cycle. These findings suggested that the increased succinylation level in NADP-ME may lead to more CO_2_ provided by Wh parts. And the CO_2_ provided by the Wh parts might be used in the Gr parts, leading to maintain the growth of chimeric leaves of *Ananas comosus* var. *bracteatus*.

The Calvin cycle is one of the CO_2_ assimilation pathways, and the hallmark of Calvin cycle is the conversion CO_2_ into carbohydrates [37]. In this study, seven succinylated enzymes were found in Calvin cycle (Fig. 9). Furthermore, four enzymatic proteins with downregulated Ksu sites were found in the Wh parts of chimeric leaves, such as the RuBisCO large chain and small chain, phosphoglycerate kinase (PGK), transketolase (TK). Importantly, RuBisCO possessed 15 and 5 succinylated sites in the large chain and small chain, respectively. RuBisCO catalyzes the limiting step of photosynthetic capacity, which plays central roles in Calvin cycle [38]. In this study, the activity of RuBisCO is inversely proportional to succinylation level of RuBisCO (*P* < 0.05; Additional file 7: Figure S4). Similarly, deacetylated RuBisCO can increase the activity of RuBisCO in *Arabidopsis* [39]. These results suggested lower level of succinylation in RuBisCO may lead to lower photosynthetic capacity in the Wh parts of chimeric leaves, through increasing activity of RuBisCO to adding content of starch. Conversely, the succinylation level of all modification sites in fructose-bisphosphate aldolase (ALDO), sedoheptulose-1,7-bisphosphatase (SBPase), glyceraldehyde-3-phosphate dehydrogenase 2 (GAPD2), and phosphoribulokinase (PRK) were upregulated in the Wh parts of chimeric leaves. Interestingly, the protein levels of GAPD2, SBPase and ALDO were downregulated in the Wh parts of chimeric leaves, indicating that the increased succinylation levels were not simply due to the increased protein levels. Our results suggested that the increase (decrease) of succinylation level of certain protein involved in the Calvin cycle may lead to higher starch content and lower soluble sugar content in the Wh parts of chimeric leaves. Therefore, the Wh parts of chimeric leaves may act as photosynthetic product sink and carbon source to enhance the photosynthetic rate of chimeric leaves.

### Succinylated enzymes involved in glycolysis, CAC and pyruvate metabolism in the Gr and Wh parts of chimeric leaves

**Fig. 10:**
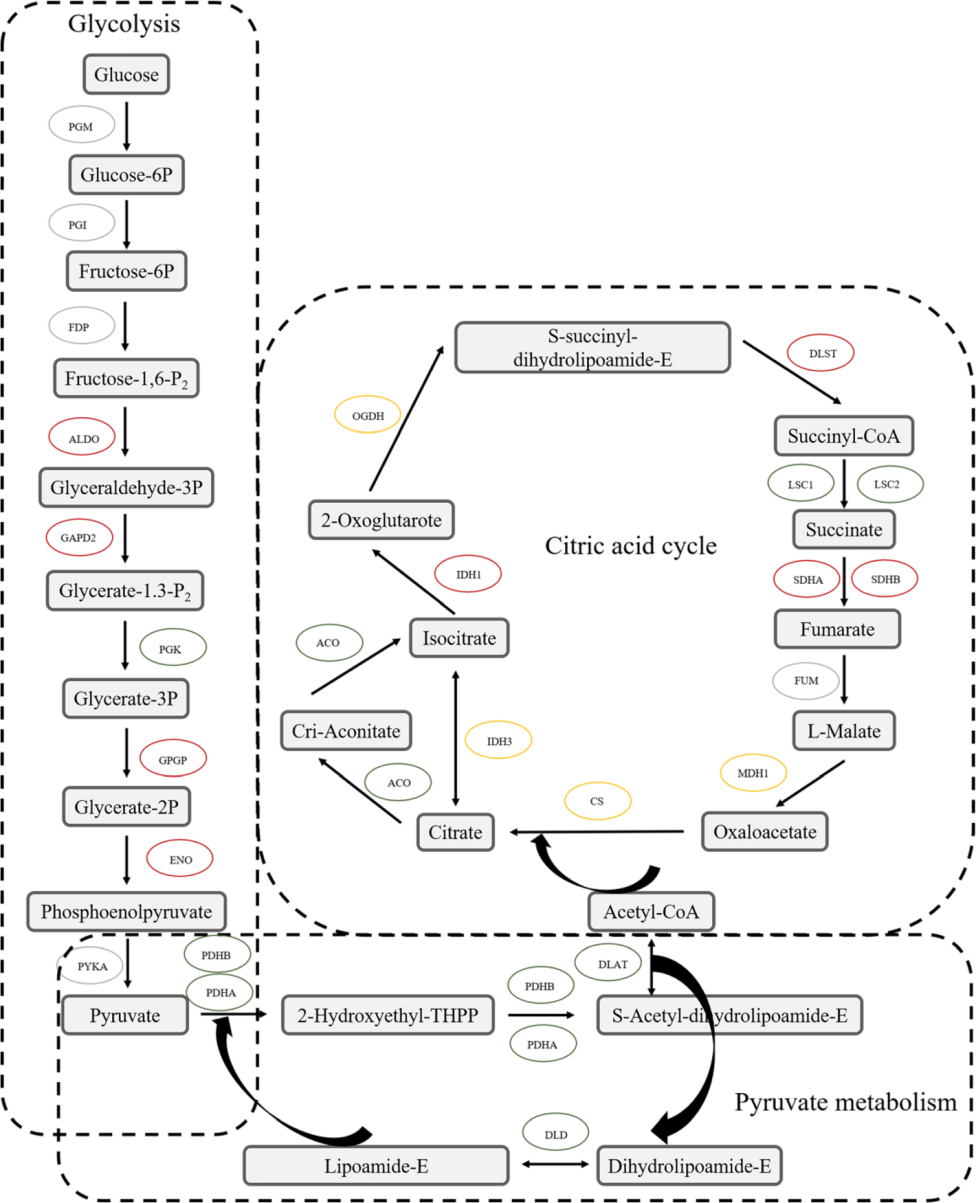
Succinylated enzymes involved in glycolysis, the CAC, and pyruvate metabolism. Red oval represents proteins possessed upregulated sites in the white (Wh) parts, green oval represents proteins possessed down regulated sites in the white (Wh) parts, yellow oval represents proteins both possessed upregulated and downregulated sites in the white (Wh) parts, grey oval represents proteins with no Ksu site

The respiratory, including glycolysis, ETC and CAC, plays crucial roles in organism survival by supplying energy to various cellular functions [40]. Most glycolytic enzymes that catalyzes the conversion of glucose to pyruvate were identified as succinylated proteins in bacteria, mammals and plants [29]. In *Ananas comosus* var. *bracteatus*, five succinylated proteins were quantified in both the Wh and Gr parts of chimeric leaves (Fig. 10). Among these proteins, the succinylation level of all modification sites in 2,3-bisphosphoglycerate-independent phosphoglycerate mutase (GPGP), enolase (ENO), GAPD2, and ALDO were upregulated in the Wh parts of chimeric leaves. Only the succinylation level of PGK was downregulated in the Wh parts of chimeric leaves. The increased acetylation level in bacteria GAPD promoted glycolysis but suppressed gluconeogenesis, indicating that acetylation level of GAPD can control the direction of glycolysis versus gluconeogenesis [41]. However, the function of succinylation in GAPD remains unknown.

The conversion of pyruvate to acetyl-CoA was catalyzed by pyruvate dehydrogenase complex (PDHC) [42]. Three components of PDHC were succinylated in both Wh and Gr parts of chimeric leaves, namely, E1 component alpha subunit (PHDA), E1 component beta subunit (PDHB), dihydrolipoyl transacetylase (DLAT) and dihydrolipoyl dehydrogenase (DLD). And the downregulated sites were identified in the Wh parts (Fig. 10).

In *Ananas comosus* var. *bracteatus*, ten enzymes in the CAC were succinylated in both Wh and Gr parts of chimeric leaves, and most of these enzymes had more than one Ksu site (Fig. 10). Among these proteins, the succinylation level of all modification sites in aconitate hydratase (ACO), succinyl-CoA synthetases (LSC1 and LSC2), and succinate dehydrogenase (SDHA) were downregulated in the Wh parts of chimeric leaves. Conversely, isocitrate dehydrogenase (IDH1) and dihydrolipoamide succinyltransferase (DLST) possessed downregulated sites in the Wh parts of chimeric leaves. In addition, citrate synthase (CS), OGDH and malate dehydrogenase (MDH1) not only had downregulated sites but also upregulated sites in the Wh parts of chimeric leaves. IDH1 is the rate-limited enzyme in the CAC [43]. In this study, two Ksu sites (K64 and K217) on IDH1were identified. Using mutagenesis-based analysis, succinylation can directly affect IDH1 activity in *E. coli* [4].

In addition to regulating CAC, α-KGDH complex also mediates succinylation either by enzymatic manner or by non-enzymatic manner [3]. In this study, E1K (OGDH), and E2K (DLST) of α-KGDH complex were succinylated. These results suggested that the different succinylated level in α-KGDH complex between the Wh and Gr parts of chimeric leaves might become a crucial regulation point. It not only regulates respiration pathway but also the entire cellular energy metabolism in chimeric leaves of *Ananas comosus* var. *bracteatus*.

### Succinylated enzymes involved in photorespiration in the Gr and Wh parts of chimeric leaves

**Fig. 11:**
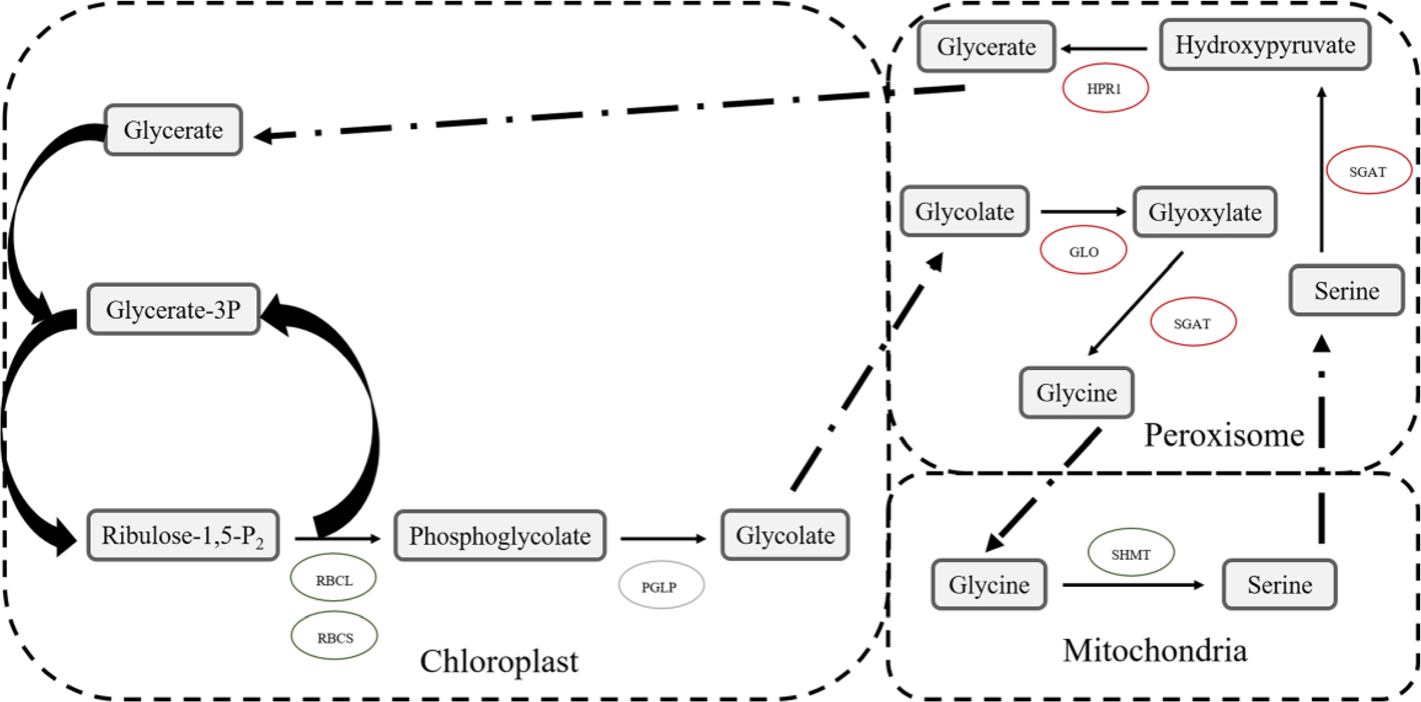
Succinylated enzymes involved in photorespiration. Red oval represents proteins possessed upregulated sites in the white (Wh) parts, green oval represents proteins possessed down regulated sites in the white (Wh) parts, yellow oval represents proteins both possessed upregulated and downregulated sites in the white (Wh) parts, grey oval represents proteins with no Ksu site

Photorespiration is a complex metabolic process in which green plants consume O_2_ under light and release CO_2_. In our study, the succinylation level of three succinylated enzymatic proteins were upregulated in the Wh parts of chimeric leaves, including peroxisomal (S)-2-hydroxy-acid oxidase (GLO), glycerate dehydrogenase (HPR1), and serine-glyoxylate aminotransferase (SGAT) (Fig. 11). Conversely, the succinylation level of all modification sites in serine hydroxymethyltransferase (SHMT), the large chain and small chain of RuBisCO were downregulated in the Wh parts of chimeric leaves. Studies have demonstrated that photorespiration not only involves in primary metabolism, including energy metabolism, amino acid synthesis and redox signaling, but also participates in plant resistance to biotic and abiotic stress [44–46]. Therefore, the lysine succinylation level may affect photorespiration capacity and ultimately lead to a difference in resistance between the Gr and the Wh parts of chimeric leaves. In addition, phosphorylation, acetylation and ubiquitination were identified in the photorespiration pathway in other plants [47–49]. However, as with succinylation, how other PTMs regulate the functions of enzymes in the photorespiration pathway has not been reported.

## Conclusions

Compared to Gr parts, the global succinylation level increased in the Wh parts of chimeric leaves according to the Western blot and immunohistochemistry analysis. Furthermore, we quantitated the change in the succinylation profiles between the Wh parts and Gr parts of chimeric leaves using label-free LFQ intensity. In total, 855 succinylated sites in 335 proteins were identified, and 593 succinylated sites in 237 proteins were quantified. Compared to the Gr parts, 232 (61.1%) sites in 128 proteins were quantified as upregulated targets, and 148 (38.9%) sites in 70 proteins were quantified as downregulated targets in the Wh parts of chimeric leaves using a 1.5-fold threshold (*P* < 0.05). These proteins with altered succinylation level were mainly involved in CAM photosynthesis, photorespiration, glycolysis, the CAC and pyruvate metabolism. Our results suggested that the changed succinylation level in proteins might function in the main energy metabolism pathways—photosynthesis and respiration. Succinylation might provide a significant effect in the growth of chimeric leaves and the relationship between the Wh and Gr parts of chimeric leaves. This study not only provided a basis for further characterization on the function of succinylated proteins in chimeric leaves of *Ananas comosus* var. *bracteatus* but also provided a new insight into molecular breeding for leaf color chimera.

## Method

### Plant materials

*Ananas comosus* var. *bracteatus* plants used in the following experiments were identified by professor He Yehua, who is an expert in pineapple classification. Three years old *Ananas comosus* var. *bracteatus* plants were cultivated from crowns. Two samples include the Wh and Gr parts of chimeric leaves (Additional file 1: Figure S1), and each sample was collected with three biological replicates.

### Protein extraction and Western blot

The total proteins extraction from sample was performed as previously described, with minor modification [26]. Briefly, the sample powder which was ground by liquid nitrogen was sonicated three times in lysis buffer (8 M urea, 1% Triton X-100, 65 mM dithiothreitol (DTT), 50 mM nicotinamide, 3 μM trichostatin A (TSA) and 1% protease inhibitor cocktail). After centrifugation at 20,000 g at 4 °C for 10 min, the supernatant combined with cold 15% trichloroacetic acid (TCA) was set at − 20 °C for 2 h. Then, the remaining precipitate obtained after centrifugation at 4 °C for 10 min was washed three times with cold acetone. The protein was redissolved in buffer (8 M urea, 100 mM NH_4_HCO_3_, pH 8.0).

Western blot assays were carried out with the method of Xu et al. [16]. Proteins were loaded on SDS-PAGE gel, and then transferred onto a PVDF membrane (Millipore) for Western blot. Succinyl-lysine residues and acetyl-lysine residues in proteins were detected by using pan anti-succinyl lysine antibody (PTM-419, China) and pan anti-acetyl lysine antibody, respectively. Secondary antibody (goat anti-mouse IgG (H + L), Thermo, 31,430) used was diluted at 10000 times.

### Trypsin digestion, succinylated peptides enrichment, and LC-MS/MS analysis

Trypsin-based method was used to digest protein. Before digestion, protein solution was first reduced for 1 h at 37 °C with 10 mM DTT. Proteins then were alkylated at room temperature in darkness with 20 mM iodoacetamide (IAA) for 45 min. Afterward, a two-step trypsin digestion was performed as previously described [16].

To enrich succinylated peptides, tryptic peptides dissolved in NETN buffer (100 mM NaCl, 1 mM EDTA, 50 mM Tris-HCl, 0.5% NP-40, pH 8.0) were incubated overnight with prewashed antibody beads (PTM Biolabs) at 4 °C. After washing four times with NETN buffer and twice with ddH_2_O, the bound peptides were eluted from the beads with 0.1% TFA.

After cleaning with C18 ZipTips (Millipore), the enriched peptides were analyzed following the procedure as described, with minor modification [16]. In brief, the peptides dissolved in 0.1% formic acid (FA) were firstly separated using a reversed-phase analytical column (Acclaim PepMap RSLC, Thermo Scientific) at a constant flow rate of 700 nl/min on EASY-nLC 1000 UPLC system. Then, the resulting peptides were subjected to MS/MS by an Orbitrap Fusion mass spectrometer (ThermoFisher Scientific). Detection of intact peptides and ion fragments in the Orbitrap were carried out at a resolution of 60,000 and 15,000, with the NCE setting as 35. For MS scans, the m/z scan range was 350 to 1550.

### Database search

The resulting MS/MS data were processed using the Maxquant search engine (v.1.5.2.8). Tandem mass spectra were searched against the *Ananas comosus* var. *bracteatus* database concatenated with a reverse decoy database. The detailed parameters used for MaxQuant are as previously described [16]. Label-free quantification method was LFQ, the false discovery rate (FDR) was adjusted to < 1% and minimum score for modified peptides score was set > 40.

### Bioinformatics analysis

Using the UniProt-GOA database (www. http://www.ebi.ac.uk/GOA/), GO annotation was performed to analyze succinylated proteins in this study. The subcellular localization of succinylated proteins was predicted by WoLF PSORT soft. And KEGG database was used to annotate protein pathway. To identify the conserved motifs, soft Motif-x was used to analyze sequence models composed of amino acids at specific positions of modified 21-mers (10 amino acids upstream and downstream of the site) from all protein sequences. Enrichment analysis of GO annotation and KEGG pathway were performed a two-tailed Fisher’s exact test. Correction for multiple hypothesis testing was carried out using standard false discovery rate control methods. Any terms with a corrected *p*-value < 0.05 was considered significant.

### Enzyme assay

RuBisCO, MDH1, NADP-ME and PEPC activity were measured as previous studies described [39, 50]. Experiments were repeated in triplicate. Statistical analysis was performed by Excel 2019 and SPSS 22.0 version. T-test at the 5% level was employed for comparing the means of two parts.

### Starch, malate and soluble sugar content analysis

Starch, malate and soluble sugar content were measured as Baldicchi et al. [51]. The statistical analysis is the same as “Enzyme assay”.

### Immunohistochemistry

At room temperature, fresh tissue sections obtained by hand-sliced method were incubated with 3% H_2_O_2_ for 10 min and blocking buffer for 20 min (Solarbio, China). After that, sections were incubated at 20 °C for 2 h with primary antibody (PTM-419, China) which was diluted 1000 times. Then, sections were incubated at 37 °C for 30 min with secondary antibody (goat anti-mouse IgG, Solarbio, China) which was diluted 100 times. The negative control that sections were incubated with PBS instead of anti-succinyllysine antibodies was set. Using the SABC-POD Kit (Solarbio, China) and light microscope, immunoreaction products were visualized and imaged.

## Supplementary information

**Additional file 1: Figure S1.** Phenotype and transverse section of chimeric leaves of *Ananas comosus* var*. bracteatus*. (**a**) Phenotype of chimeric leaves of *Ananas comosus* var. *bracteatus*. A: potted plant of *Ananas comosus* var*. bracteatus*; B: green parts of chimeric leaves; C: white parts of chimeric leaves. (**b**) Transverse section of chimeric leaves of *Ananas comosus* var. *bracteatus*. A, B, C, D represent the different parts of the chimeric leaves, respectively. Scale bar = 1 cm and 200 μm (in b).

**Additional file 2: Figure S2.** Western blot analysis of the protein posttranslational modification levels between the white (Wh) parts and green (Gr) parts chimeric leaves of *Ananas comosus* var. *bracteatus*. **a**, **b** Western blot of protein acetylation (**a**), succinylation (**b**). **c** SDS-PAGE stained with coomassie blue. Same amount of proteins (30 μg per lane) were loaded as in each panel. The Western blot experiment of each part of chimeric leaves is repeated three times.

**Additional file 3: Table S1.** Characteristics of the 1262 proteins differentially expressed between the white (Wh) parts and green (Gr) parts of chimeric leaves of *Ananas comosus* var. *bracteatus*.

**Additional file 4: Figure S3.** KEGG  pathway-based enrichment analysis of up-regulated (**a**) and down-regulated (**b**) proteins in the white (Wh) parts.

**Additional file 5: Table S2.** Proteins with opposing changes of protein and succinylation levels in the white (Wh) parts. Gr: green parts.

**Additional file 6: Table S3.** Characteristics of the 380 lysine succinylation sites differentially expressed between the white (Wh) parts and green (Gr) parts of chimeric leaves of *Ananas comosus* var. *bracteatus*.

**Additional file 7: Figure S4.** Enzymatic assays showed that the activity of MDH (**a**), NADP-ME (**b**), PEPC (**c**), and Rubisco (**d**) between the two parts of chimeric leaves of *Ananas comosus var*. *bracteatus*. Standard error of the mean for three repetitions is represented by the error bars. The different letters above the bars indicate the significant difference at *P* < 0.05 between two parts. Wh: white parts; Gr: green parts.

## Data Availability

The datasets supporting the results of this article are included within the article and supplementary information.

## Abbreviations

ACN: Acetonitrile
ACO: Aconitate hydratase
AGC: Automatic gain control
ALDO: Fructose-bisphosphate aldolase
CAC: Citric acid cycle
CAM: Crassulacean acid metabolism
CS: Citrate synthase
DEPS: Differentially expressed proteins
DGS: Differentially expressed unigenes
DLAT: Dihydrolipoyl transacetylase
DLD: Dihydrolipoamide dehydrogenase
DLST: Dihydrolipoyl transacetylase
DTT: Dithiothreitol
ENO: Enolase
FA: Formic acid
FDR: False discovery rate
GAPD2: Glyceraldehyde-3-phosphate dehydrogenase 2
GLO: Peroxisomal (S)-2-hydroxy-acid oxidase
GO: Gene Ontology
GPGP: 2,3-bisphosphoglycerate-independent phosphoglycerate mutase
Gr: Green parts
HPR1: Glycerate dehydrogenase
IAA: Iodoacetamide
IDH1: Isocitrate dehydrogenase
IDH3: Isocitrate dehydrogenase (NAD+)
KEGG: Kyoto encyclopedia of genes and genomes
KGDHC: α-ketoglutarate dehydrogenase complex
LFQ: Label-free quantification
LHCB2: Light-harvesting complex II chlorophyll a/b binding protein 2
LSC1: Succinyl-CoA synthetase 1
LSC2: Succinyl-CoA synthetase 2
LS-MS/MS: Liquid chromatography-tandem mass spectrometry
MDH1: Malate dehydrogenase1
NADP-ME: NAD-dependent malic enzyme
OGDH: 2-oxoglutarate dehydrogenase
PDHA/B: E1 component alpha/beta subunit of Pyruvate dehydrogenase
PEPC: Phosphoenolpyruvate carboxylase
PGK: Phosphoglycerate kinase
PPIs: protein-protein interactions
PRK: Phosphoribulokinase
PTM: Protein posttranslational modification
RBCL/S: Ribulose bisphosphate carboxylase large chain/small chain
SBPase: Sedoheptulose-1,7-bisphosphatase
SDHA/B: Succinate dehydrogenase
SGAT: Serine-glyoxylate aminotransferase
SHMT: Serine hydroxymethyltransferase
TCA: Trichloroacetic acid
TK: Transketolase
TSA: Trichostatin A
Wh: White parts
