# Supplementary figures and images for: Systematic identification and comparative analysis of lysine succinylation between the green and white parts of chimeric leaves of Ananas comosus var. bracteatus
Source: BMC Genomics. 2020 Jun 3;21:383. doi: 10.1186/s12864-020-6750-6 (PMC7268518; doi:10.1186/s12864-020-6750-6)

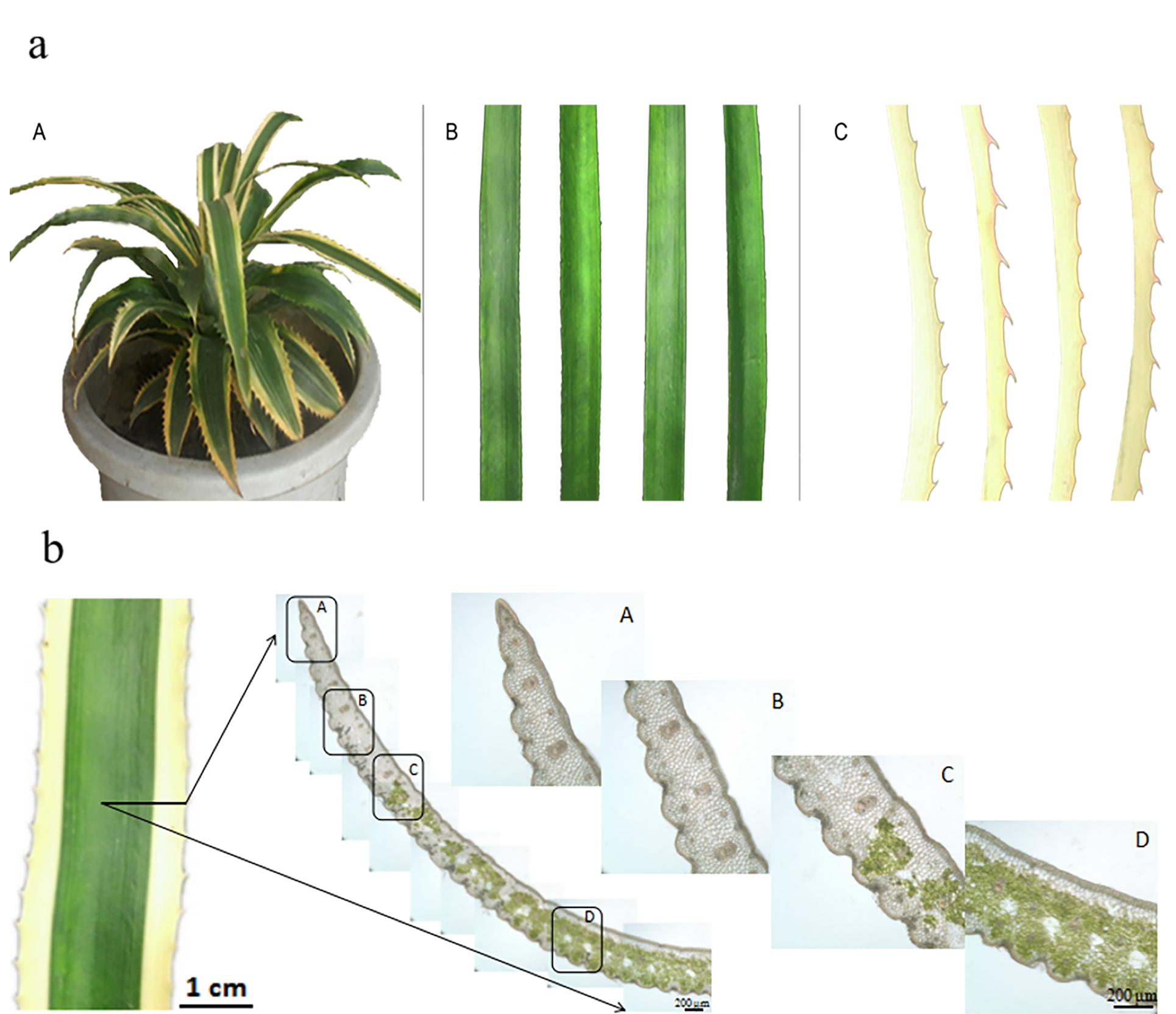

Supplement: Supplementary file 1 — Additional file 1: Figure S1. Phenotype and transverse section of chimeric leaves of Ananas comosus var. bracteatus. (a) Phenotype of chimeric leaves of Ananas comosus var. bracteatus. A: potted plant of Ananas comosus var. bracteatus; B: green parts of chimeric leaves; C: white parts of chimeric leaves. (b) Transverse section of chimeric leaves of Ananas comosus var. bracteatus. A, B, C, D represent the different parts of the chimeric leaves, respectively. Scale bar = 1 cm and 200 μm (in b). [file 12864_2020_6750_MOESM1_ESM.tif]

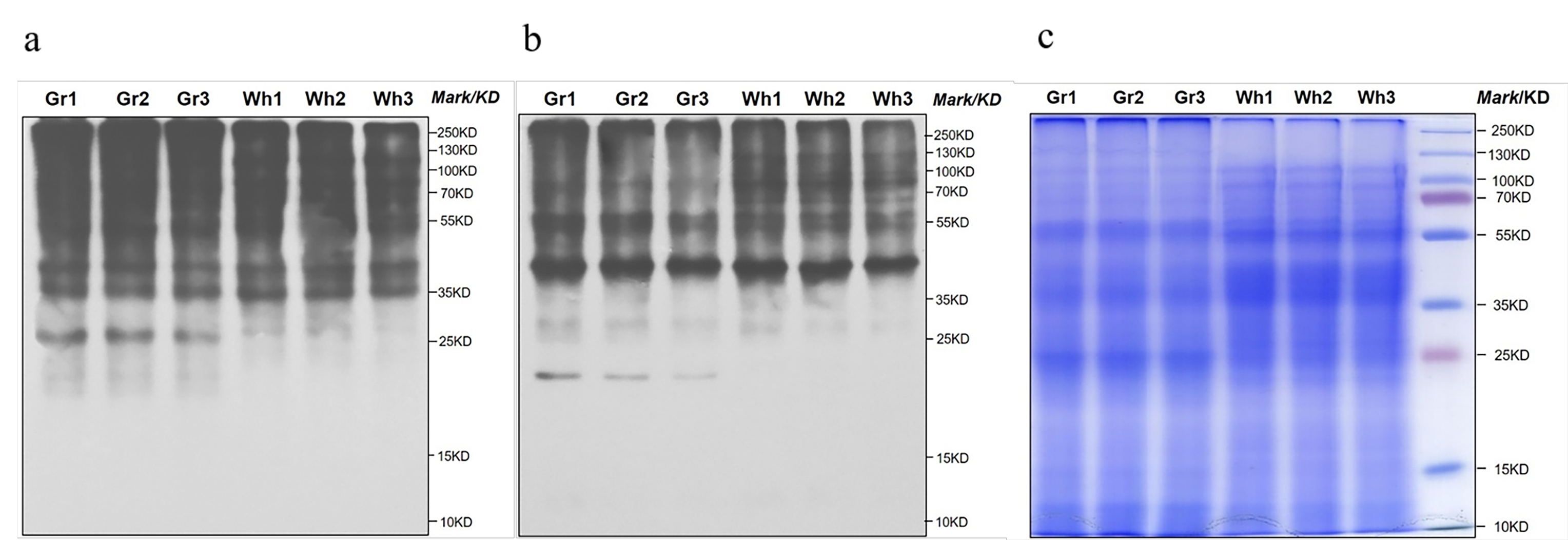

Supplement: Supplementary file 2 — Additional file 2: Figure S2. Western blot analysis of the protein posttranslational modification levels between the white (Wh) parts and green (Gr) parts chimeric leaves of Ananas comosus var. bracteatus. a, b Western blot of protein acetylation (a), succinylation (b). c SDS-PAGE stained with coomassie blue. Same amount of proteins (30 μg per lane) were loaded as in each panel. The Western blot experiment of each part of chimeric leaves is repeated three times. [file 12864_2020_6750_MOESM2_ESM.tif]

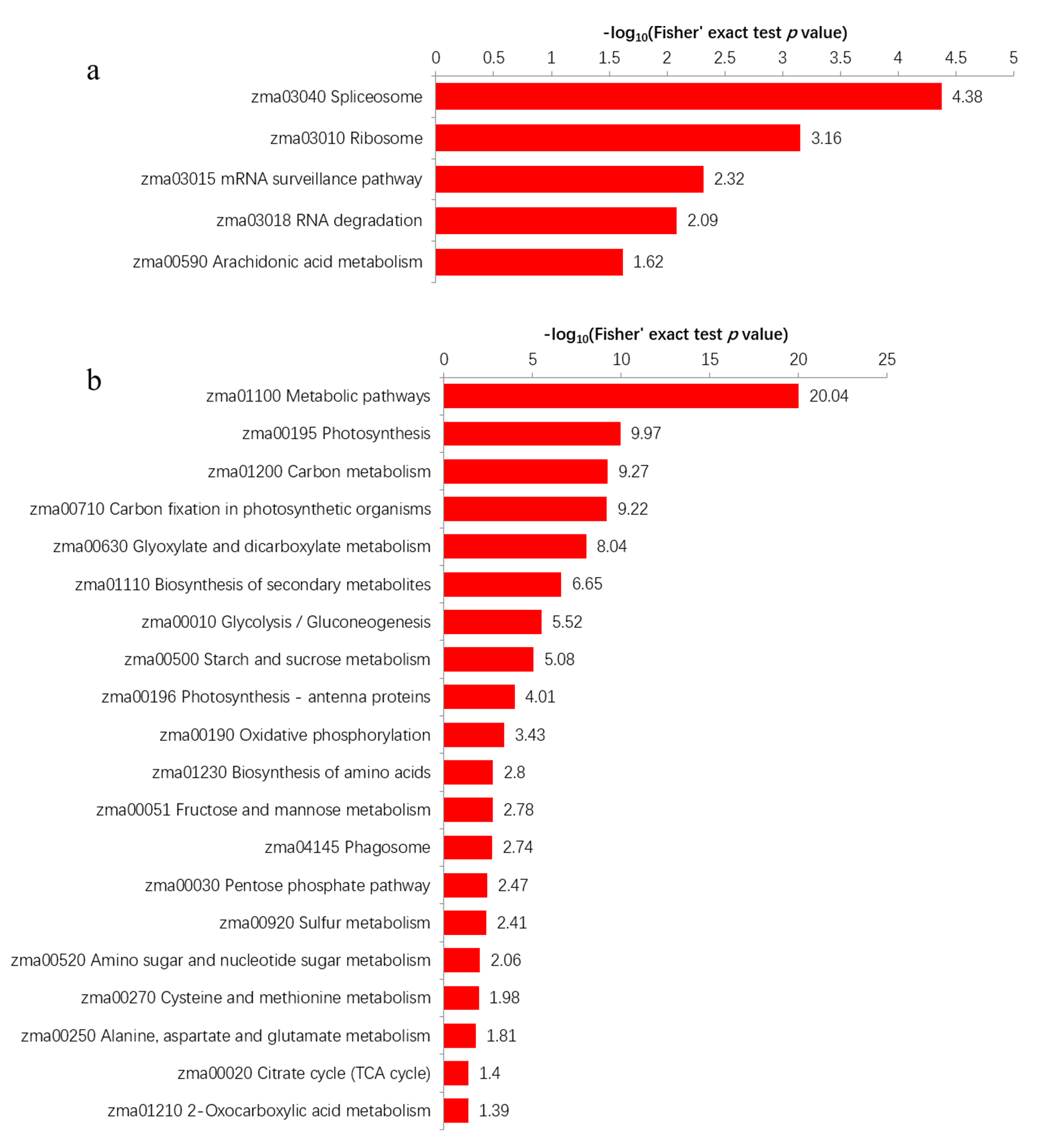

Supplement: Supplementary file 4 — Additional file 4: Figure S3. KEGG pathway-based enrichment analysis of up-regulated (a) and down-regulated (b) proteins in the white (Wh) parts. [file 12864_2020_6750_MOESM4_ESM.tif]

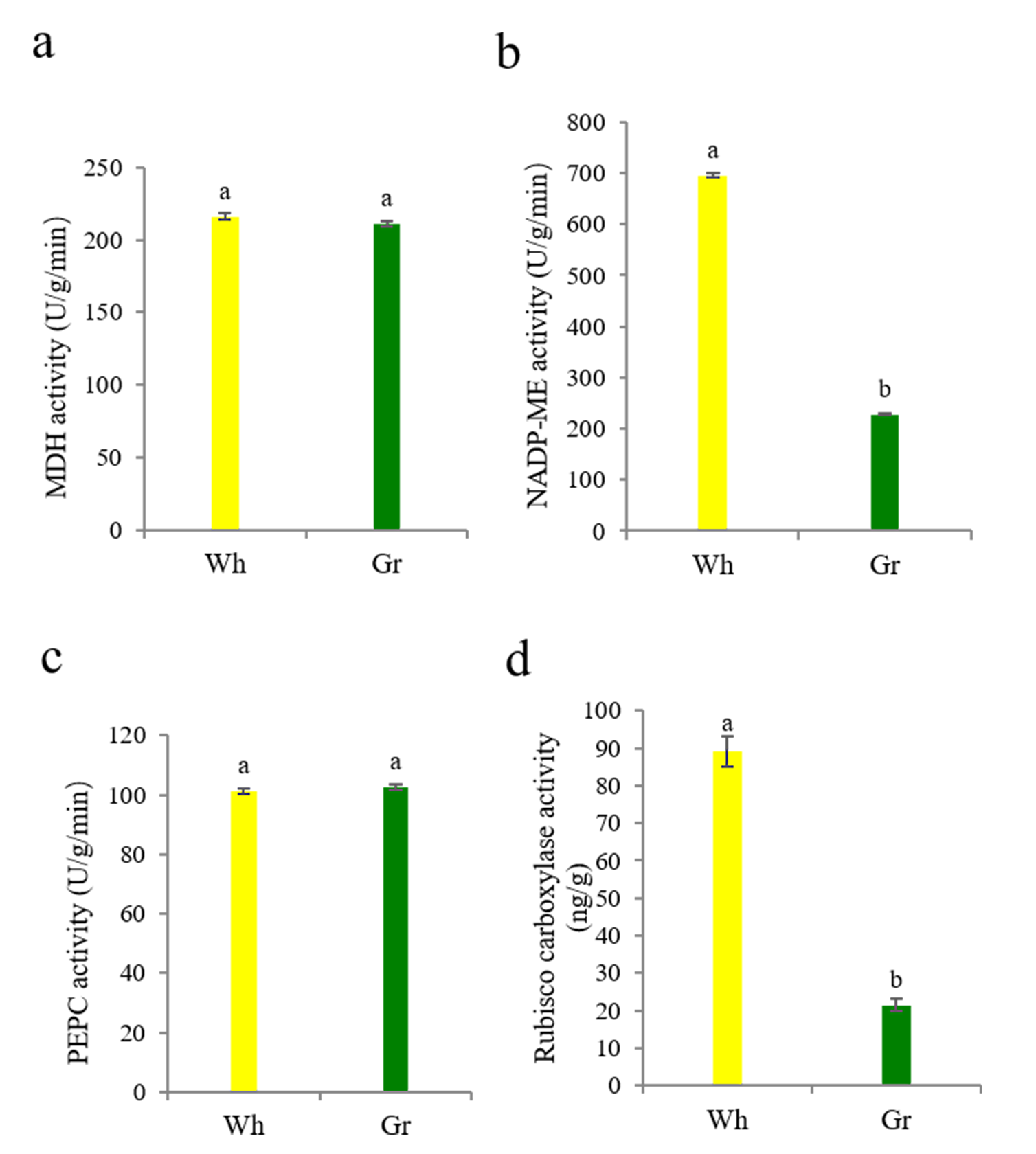

Supplement: Supplementary file 7 — Additional file 7: Figure S4. Enzymatic assays showed that the activity of MDH (a), NADP-ME (b), PEPC (c), and Rubisco (d) between the two parts of chimeric leaves of Ananas comosus var. bracteatus. Standard error of the mean for three repetitions is represented by the error bars. The different letters above the bars indicate the significant difference at P < 0.05 between two parts. Wh: white parts; Gr: green parts. [file 12864_2020_6750_MOESM7_ESM.tif]
